# Overview of mechanism of electroacupuncture pretreatment for prevention and treatment of cardiovascular and cerebrovascular diseases

**DOI:** 10.1111/cns.14920

**Published:** 2024-10-03

**Authors:** Jiaming Zeng, Jiaojiao Cao, Haitao Yang, Xue Wang, Tingting Liu, Zhihan Chen, Fangyuan Shi, Zhifang Xu, Xiaowei Lin

**Affiliations:** ^1^ Research Center of Experimental Acupuncture Science, School of Acupuncture‐Moxibustion and Tuina Tianjin University of Traditional Chinese Medicine Tianjin China; ^2^ Tianjin Key Laboratory of Modern Chinese Medicine Theory of Innovation and Application, School of Traditional Chinese Medicine Tianjin University of Traditional Chinese Medicine Tianjin China; ^3^ National Clinical Research Center for Chinese Medicine Acupuncture and Moxibustion Tianjin China

**Keywords:** cardiovascular disease, cerebrovascular diseases, electroacupuncture pretreatment, mechanism, meridian

## Abstract

Cardio‐cerebrovascular disease (CCVD) is a serious threat to huma strategy to prevent the occurrence and development of disease by giving electroacupuncture intervention before the disease occurs. EAP has been shown in many preclinical studies to relieve ischemic symptoms and improve damage from ischemia–reperfusion, with no comprehensive review of its mechanisms in cardiovascular disease yet. In this paper, we first systematically discussed the meridian and acupoint selection law of EAP for CCVD and focused on the progress of the mechanism of action of EAP for the prevention and treatment of CCVD. As a result, in preclinical studies, AMI and MCAO models are commonly used to simulate ischemic injury in CCVD, while MIRI and CI/RI models are used to simulate reperfusion injury caused by blood flow recovery after focal tissue ischemia. According to the meridian matching rules of EAP for CCVD, PC6 in the pericardial meridian is the most commonly used acupoint in cardiovascular diseases, while GV20 in the Du meridian is the most commonly used acupoint in cerebrovascular diseases. In terms of intervention parameters, EAP intervention generally lasts for 30 min, with acupuncture depths mostly between 1.5 and 5 mm, stimulation intensities mostly at 1 mA, and commonly used frequencies being low frequencies. In terms of molecular mechanisms, the key pathways of EAP in preventing and treating cardiovascular and cerebrovascular diseases are partially similar. EAP can play a protective role in cardiovascular and cerebrovascular diseases by promoting autophagy, regulating Ca^2+^ overload, and promoting vascular regeneration through anti‐inflammatory reactions, antioxidant stress, and anti‐apoptosis. Of course, both pathways involved have their corresponding specificities. When using EAP to prevent and treat cardiovascular diseases, it involves the metabolic pathway of glutamate, while when using EAP to prevent and treat cerebrovascular diseases, it involves the homeostasis of the blood–brain barrier and the release of neurotransmitters and nutritional factors. I hope these data can provide experimental basis and reference for the clinical promotion and application of EAP in CCVD treatment.

## Graphical abstract

The green arrow represents the mechanism of EAP GV20 acupoint in preventing and treating cerebrovascular diseases. The red arrow represents the mechanism of EAP PC6 acupoint in preventing and treating cardiovascular diseases.

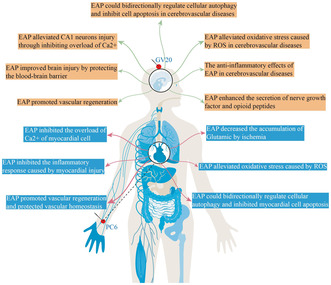

## 1 INTRODUCTION

With the improvement of people's living standards, the acceleration of life pace, and the increase of life pressure, Cardio‐cerebrovascular‐disease (CCVD) has become highly prevalent chronic diseases. CCVD is a general term for a series of ischemic or hemorrhagic CCVD of the heart and brain due to blood viscosity, atherosclerosis, hyperlipidemia, and high blood pressure.^1^ Not only brings physical pain and even threaten life but also brings great mental stress, suffering from both physical and psychological torment, and causing losses in multiple aspects.^2^ Currently, the main clinical treatments are medication, interventional therapy, surgical treatment, etc. Although these treatments are beneficial to the reduction of symptoms, they may also produce drug dependence as well as side effects. The etiology of CCVD is complex and includes biological, genetic, environmental factors, and poor lifestyle, especially unhealthy lifestyle habits that are important triggers of cardiovascular disease.^3^ Finding treatments to prevent the occurrence or exacerbation of CCVD is the key to reducing the cardiovascular threat.

The essence of the theory of “Chinese medicine treating the unhealthy” is to emphasize the prevention of illness before it occurs. Electroacupuncture pretreatment (EAP) therapy refers to a kind of treatment method in which an electric needle is inserted into a specific acupoint of the human body and weak current stimulation is given before the disease occurs or worsens, so as to achieve the purpose of treating disease and health care. A large number of modern preclinical studies have found that EAP intervention before disease onset can reduce myocardial infarction size and myocardial cell damage caused by ischemia.^4^ In addition, EAP can also promote vasodilation and increase blood flow, thereby improving brain microcirculation and reducing brain tissue damage caused by brain hypoxia and insufficient energy supply.^5^ In addition to the efficacy of EAP in the treatment of CCVD, a large number of scholars have also conducted a series of studies on the scientific mechanism of EAP in the treatment of CCVD.^6^ Therefore, this paper reviewed the relevant studies on EAP treatment of CCVD in the past 20 years and systematically reviewed the research progress in multiple aspects of EAP intervention acupoint selection rule, acupuncture parameters, and its mechanism of action, in order to lay a foundation for the clinical application and promotion of EAP treatment of CCVD.

## 2 METHOD

### 2.1 Search strategy

Using the PubMed database, we searched for studies published between January 2003 and 2024, using medical subject titles [“electroacupuncture preconditioning” or “electroacupuncture pretreatment” and “cardiovascular disease” or “cerebrovascular disease” or “myocardial reperfusion injury” or “myocardial ischemia–reperfusion” or “myocardial ischemia‐ reperfusion injury” or “myocardial ischemia” or “cerebral ischemia‐reperfusion injury” or “cerebral ischemia reperfusion injury” or “cerebral ischemic injury”] as keywords, Cardiovascular and cerebrovascular disease is a general term for cardiovascular and cerebrovascular diseases, and no other synonyms have been found. The language is limited to English and Chinese. A total of 182 articles were selected.

### 2.2 Study selection and data extraction

**FIGURE 1:**
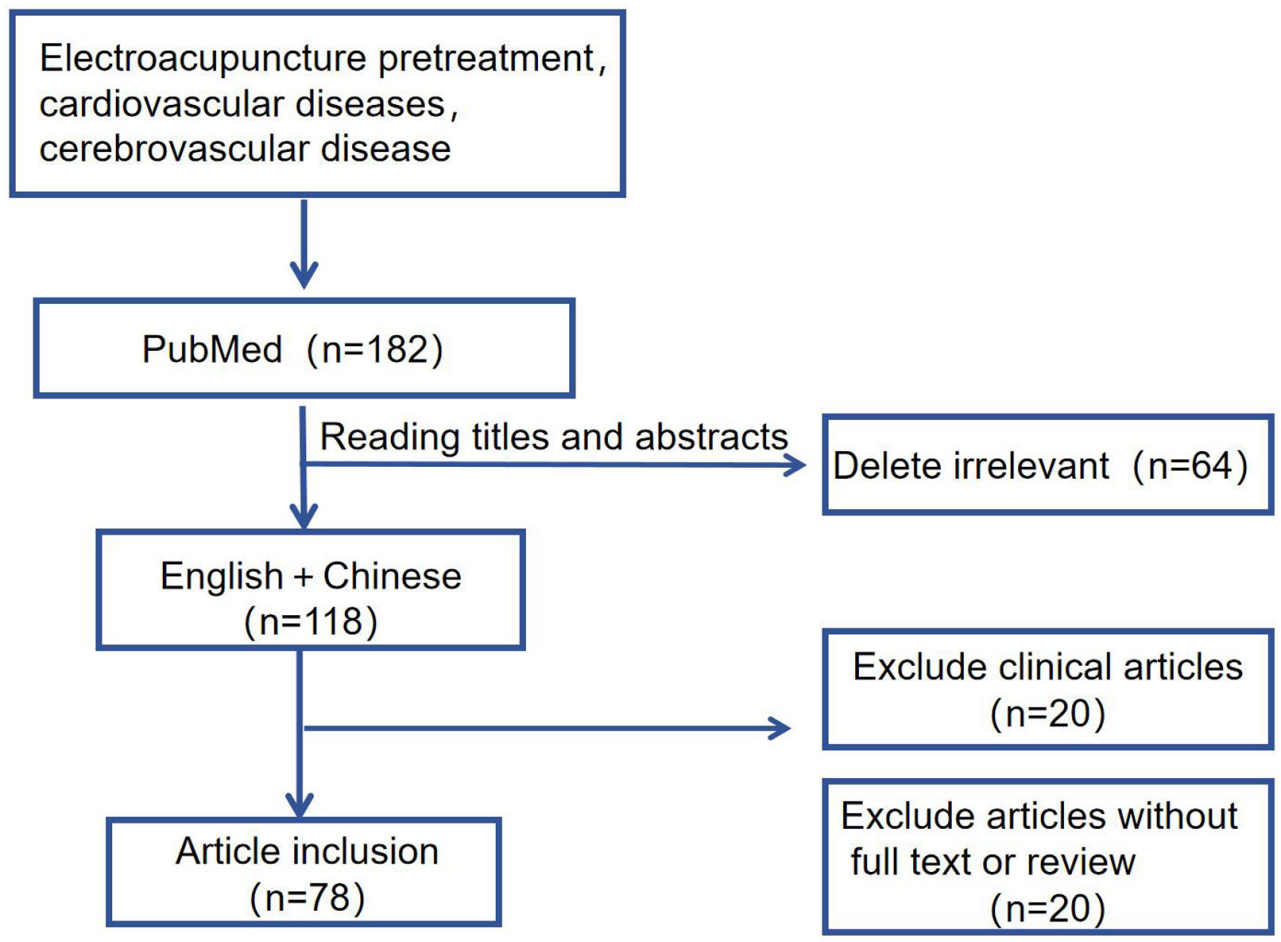
A flowchart of this search process.

In the remaining 118 articles, we excluded the articles without full text, the remaining articles with full text were manually screened, and finally included 78 basic research articles. The flow diagram of this search process is shown in Figure 1.

In addition, we extracted the relevant information of these 78 literatures, including the animal models and species used in the research, parameter of acupuncture, intervention acupoints, detection tissues, and molecules and arranged them in Table 1. The data are extracted by one author and proofreading by other authors.

**TABLE 1:** Parameters, sepsis model, acupoints, and detection indexes of EAP in prevention and treatment of CCVD.

Sepsis model	Intervention methods	Species	Parameter of acupuncture	Acupoints	Test site Site + Mechanism	References
MIRI	EAP	Rat	2/15 Hz, 1 mA, 30 min/d, 3 d	PC6	Heart: TRH↓, Myh7b↓, MyI3↓, Tnni3↑, Actc1↑, Cacna1c↑	Huang (2014)^207^
SGIR	EAP	Rat	20 Hz, 1–3 mA, 30 min/d, 3 d	PC6	Ventricular tissue: Cx43↓	Gao (2015)^77^
MCAO	EAP	Rat	2/50 Hz, 30 min/d, 5 d	GV20, BL23, SP6	Hippocampus: TPPV1↓	Long (2022)^133^
MIRI	EAP	Rat	2 Hz, 1 mA, 20 min/d, 7 d	HT7, HT5	LHA: GABA↑, GLU↓	Yu (2021)^86^
CI/RI	EAP	Rat	2/50 Hz, 1 mA, 30 min/d, 5 d	GV20, GV26	Peri‐infarct cortex: LC3‐II↑, p‐mTORC1↓	Tian (2022)^128^
MCAO	EAP	Mice	2/15 Hz, 1 mA, 30 min/d, 7 d	GV20	Penumbral astrocyte: eCBs↑	Yang (2021)^148^
MIRI	EAP	Rat	2/15 Hz, 30 min/d, 5 d	PC6	Serum: CK‐MB↓, INOS↓, T‐AOC↑	Han (2021)^53^
AMI	EAP	Mice	2/15 Hz, 2 mA, 20 min/d, 3 d	PC6	Heart: IL‐Iβ↓, NLRP3↓, IL‐1↓	Zhang (2020)^24^
MCAO	EAP	Rat	2/15 Hz, 20 min/d, 5 d	GV20, PC6, SP6	Hippocampus: p38MAPK↓	Zhang (2020)^195^
MCAO	EAP	Mice	1 mA, 2/15 Hz, 30 min	PC6, ST36	Adenosine A1: P‐GSK‐3β↑	Geng (2020)^198^
MIRI	EAP	Rat	2 mA, 2/10 Hz, 30 min	PC6, ST36	Mitochondria: Bcl‐2↑, ROS↓	Wang (2020)^44^
MCAO	EAP	Rat	1 mA, 2/15 Hz, 30 min/d, 5 d	GV20	Endothelium: p‐caveolin‐1↑, p‐Akt↑	Zou (2015)^157^
MCAO	EAP	Rat	1 mA, 2/15 Hz, 30 min/d, 2 d	GV20	Brain: McPIP1↑, TNF‐α↓, IL‐1β↓, IL‐6↓, MCP‐1↓, NeuronsMPIP1↑	Jin (2013)^200^
MCAO	EAP	Mice	1 mA, 2/15 Hz, 30 min	GV20	Neuronal: GluR2↑	Liu (2015)^139^
MCAO	EAP	Rat	1 mA, 2 /100 Hz, 1 h, 4 d	GV20, BL23, SP6	Hippocampus: TNF‐α↓, L‐1β↓, TRPV‐1↓	Long (2019)^120^
MCAO	EAP	Mice	1 mA, 2 Hz, 30 min/d, 3 d	GV20	Brain: TNF‐α↓, IL‐6↓, Heart: Nox2↓, Nox4 ↓	Yong (2019)^201^
MCAO	EAP	Mice	1 mA, 2/15 Hz, 30 min	GV20	Brain: Ros↓, MDA↓	Wang (2009)^141^
MCAO	EAP	Rat	1 mA, 2/15 Hz, 30 min/d, 5 d	GV20	Ischemic cortex: Beclin‐1↓, LC3‐II↑	Wu (2015)^127^
MCAO	EAP	Rat	1 mA, 2/15 Hz, 30 min,5 d	GV20	Brain: HMGB↑	Wang (2012)^113^
MCAO	EAP	Mice	2 /15 Hz, 20 min/d, 3 d	PC6	Myocardium: NLRP3↓, IL‐1β↓	Zhang (2020)^33^
CPR	EAP	Rat	2 mA, 2/10 Hz, 30 min	PC6, ST36, ST40	Heart: MAP↑, HR↑	Gao (2016)^43^
MCAO	EAP	Rat	1 mA, 2/15 Hz, 2.5 h	GV20	Cerebral ischemic: p‐GSK‐3β↑	Wei (2014)^173^
MCAO	EAP	Mice	1 mA, 2/15 Hz, 30 min	GV20	Neuronal: AdipoR1 ↑, p‐GSK‐3β↓	Guo (2015)^179^
AMI	EAP	Mice	0.5 mA, 1 mA, 3 mA, 2 /15 Hz, 20 min/d, 3 d	PC6	Myocardial tissue: TNF‐α↓, TLR4↓, IL‐1β ↓	Yang (2022)^25^
MCAO	EAP	Rat	1 mA, 2/15 Hz, 30 min/d, 5 d	GV20	Neuronal: HIF‐1α↑, NICD↑, Bcl‐2↑, Bax ↓	Zhao (2015)^196^
MCAO	EAP	Rat	1 mA, 2 /100 Hz, 20 min/d, 7 d	PC6, ST36, CV4	Serum: TNF‐α↓, IL‐6↓, myocardial tissue: FXR↓, SHP↓AIF↓	Liu (2021)^62^
CI/RI	EAP	Mice	1 mA, 2 Hz, 20 min/d, 3 d	GV20, GV14	Plasma: SDF‐1α↑, brain: BDNF↑	Kim (2013)^169^
MIRI	EAP	Rat	1 mA, 10/50 Hz, 20 min/d, 7 d	PC6	Myocardial tissue: IC3‐II↑	Du (2019)^132^
MCAO	EAP	Rat	1 mA, 2/15 Hz, 30 min	GV20	Brain: Bcll‐2‐Bax↑	Wang (2011)^177^
MCAO	EAP	Rat	5 /10 mA, 2/15 Hz, 30 min/d, 5 d	GV20, SP6, ST36	Brain: Nrf2↑, TNF‐α↓, microglia M1: INOS↓, M2: Arg‐1↑	Li (2023)^98^
AMI	EAP	Rat	1 mA, 2/15 Hz, 30 min/d, 14 d	PC6	Serum: LDH↓, Ampka1↑, PPK2↑	Wang (2020)^51^
MCAO	EAP	Rat	1 mA, 2/15 Hz, 30 min/d, 5 d	GV20, LI11, ST36	Cortex tissue: LC3‐II/LC3‐I↓, p62↑	Huang (2019)^129^
MIRI	EAP	Rat	10 /50 Hz, 30 min/d, 7 d	PC6, LI4	Serum: amino acid↓, glucose↑	Chen (2019)^209^
MCAO	EAP	Rat	1 mA, 2/15 Hz, 30 min/d, 5 d	GV20	Brain: neurons↑	Xiong (2007)^184^
MCAO	EAP	Rat	1 mA, 2/15 Hz, 30 min, 2 h	GV20, GV14	Cortex tissue: IL‐1β↓, IL‐6↓, TNF‐α↓	Chen (2021)^210^
MIRI	EAP	Rat	2 mA, 2 /100 Hz, 20 min/d, 5 d	PC6	Serum: CTnI↓, ULK1↓, Beclin‐1↓	Chen (2018)^58^
MCAO	EAP	Rat	2/15 Hz, 30 min/d, 5 d	GV20, GV14	Brain: GLU‐1↓	Guo (2015)^199^
MCAO	EAP	Rat	2.7 /3.0 mA, 5 Hz, 25 min/d, 2 d	GV20, GV14	Cortex tissue: PMEK1/2↑, Perk1/2↑, PP90RK↑	Cheng (2014)^168^
CI/RI	EAP	Rat	1 mA, 2/15 Hz, 30 min/d, 5 d	GV20, GV14	Hippocampus: GRP78↑, GADD153↓	Chen (2014)^136^
MIRI	EAP	Rat	10 /50 Hz, 1 mA, 20 min/d, 7d	PC6	Heart: LC3II↓, Beclin1↓, LC3II/LC3I↓	Tan (2018)^131^
MIR	EAP	Rabbit	1 mA, 10/50 Hz, 20 min/d, 5 d	PC6	Serum: CK↓, plasma: ET↓	Wang (2014)^95^
CI/RI	EAP	Rat	1 mA, 2/15 Hz, 30 min/d, 5 d	GV20	Ischemic penumbra: NgB↑	Xie (2012)^211^
MI	EAP	Rabbit	2–4 mA, 2 /70 Hz, 30 min/d, 7 d	PC6	Serum: AST↓, HD↓, CK‐MB↓, LDH↓, α‐HBD ↓	Huang (2012)^212^
VD	EAP	Rat	1 mA, 20 min/d, 10 d	GV20, BL23, ST36	Hippocampus: GLU↓, NMDAR1↓	Meng (2008)^172^
MCAO	EAP	Rat	1 mA, 2/15 Hz, 30 min/d, 7 d	GV20, GV26	Brain: VIGF↓, MMP‐9↓	Lin (2015)^159^
CI/R	EAP	Rat	1 mA, 2/15 Hz, 30 min/d, 5 d	GV20, GV14	Ischemic cortex: GRP78↓	Chen (2014)^107^
MCAO	EAP	Rat	1 mA, 2/15 Hz, 30 min, 2 h	GV20	Brain: ADA↓	Wang (2013)^143^
MI	EAP	Rat	5 mA, 20 Hz, 30 min/d, 3 d	PC6, ST36	Heart: β‐ARs↑	Gao (2006)^68^
MIR	EAP	Rat	5 mA, 20 Hz, 30 min/d, 3 d	PC6	Heart: β1‐AR↓	Gao (2007)^69^
SGIR	EAP	Rat	5 mA, 20 Hz, 30 min/d, 3 d	PC6	Heart: β1‐AR↓, Gs protein↓, cAMP↓	Gao (2008)^72^
MIRI	EAP	Rabbit	30/100 Hz, 20 min/d, 5 d	PC6	Myocardial tissue: Cx43↑	Zhou (2013)^78^
MCAO/R	EAP	Mice	4 Hz, 1.5 s, 16 Hz, 1.5 s	CV24, GV26	Brain: IL‐1β↓, IL‐6↓, TNF‐α↓	Zou (2016)^208^
CPB	EAP	Rat	1.5 mA, 2 /100 Hz, 10 min/d, 7 d	PC6, LI4	Serum: CTnI↓, LDH↓, heart: TNF‐α↓, IL‐1β↓	Wang (2020)^205^
MCAO	EAP	Mice	1 mA, 2/15 Hz, 30 min	GV20	Brain: MDA↓	Sun (2016)^176^
MIRI	EAP	Rat	1 mA, 8/80 Hz, 30 min/d, 5 d	PC6	Myocardial tissue: DOR↑	Li (2011)^216^
MIRI	EAP	Rat	1 mA, 2 Hz, 30 min/d, 7 d	HT7, HT5	Serum: NE↑, CK‐MB↑, CTnI↑	Wang (2023)^217^
MCAO	EAP	Mice	1 mA, 2/10 Hz, 30 min/d, 5 d	GV20	Neuronal: TREM2↑, LC3II/LC3I↑, Beclin1↑, p62↓	Yang (2023)^124^
AMI	EAP	Rat	2 mA, 2/100 Hz, 20 min/d, 7 d	PC6	Myocardial tissue: NF‐κB‐65↑, TRPV1/CGRP↑	Wu (2011)^30^
MCAO	EAP	Rat	2/15 Hz, 1 mA, 30 min /d, 5 d	GV20	Brain: p‐ERK1/2↓	Du (2010)^174^
CMI	EAP	Rat	2 Hz, 1 mA, 30 min/d, 3 d	PC6, GV14, ST36	Serum: VEGF↑, HIF‐1α↑	Fu (2021)^93^
MCAO	EAP	Rat	20 Hz, 1–3 mA, 10 min/d, 21 d	GV20	Brain: LC3II↓, P62↑	Chen (2020)^130^
MCAO	EAP	Rat	2 Hz, 1 mA, 30 min	GV20	Brain: CB2↑	Ma (2011)^144^
MCAO	EAP	Rat	2 Hz, 1 mA, 30 min	PC6	Hippocampus: Wnt/β‐catenin↑	He (2016)^145^
AMI	EAP	Rat	4–6 mA, 30 min/d, 7 d	PC6	Myocardial: CK‐MB↓, LDH↓, Bcl2↓, P62↓, LC3↑, p‐AMPK↓	Zeng (2018)^48^
MCAO	EAP	Rat	1 mA, 2/15 Hz, 30 min	GV20	Neuronal: p‐STAT3↑	Zhou (2013)^175^
MIRI	EAP	Mice	1 mA, 2 Hz, 20 min/d, 7 d	HT7, KI7	Serum: cTnI ↓, CK‐MB↓	Shu (2023)^203^
I/R	EAP	Rat	2 Hz, 30 min/d, 3 d	PC6	Myocardium: cAMP↓, Gsα↓	Gao (2006)^70^
MCAO	EAP	Rat	15 Hz, 30 min	GV20, GV26, GV14, GV9, GV4	Brain: SRC↑, PI3K↑	Wu (2021)^161^
MI	EAP	Mice	1 mA, 2 Hz, 30 min	PC6, ST36	Myocardium: PI3K↑, Akt↑	Wang (2016)^213^
SICM	EAP	Mice	10 Hz, 15 min	ST36	Serum:cTn‐1↓, exosome:miR‐381↑	Chen (2024)^204^
CIRI	EAP	Rat	1–2 mA, 2 Hz/5 Hz, 20 min/7 d	GV20, GV16, DU14	Cerebral cortex:ppARy↑, NLRP3↓	Tong (2023)^214^
MIRI	EAP	Rat	1.5 mA, 2 Hz, 20 min/7 d	HT7, KI7	Heart: ck‐MB↓	Zhou (2023)^85^
CIRI	EAP	Rat	1–2 mA, 12 Hz/15 Hz,20 min/7 d	GV20, GV16, DU14	Brain:Acsl4↓	Wu (2024)^215^
MIRI	EAP	Rat	1.5 mA, 2 Hz, 30 min/7 d	HT5, HT7	Brain:CRH↑,C‐Fos↓	Zhou (2024)^88^
MCAO	EAP	Rat	2‐20 Hz, 30 min, 3 d	GV20, G87	Brain:NF‐κB↓, NLRP3↓	Su (2024)^206^
MCAO	EAP	Rat	1 mA, 2/15 Hz, 30 min/10 d	GV20	Brain:miR‐155‐5P↓	Li (2023)^202^
MCAO	EAP	Rat	2 Hz/15 Hz, 12d	GV20	Brain:SOD↓,GPX4↓	Yang (2023)^197^
MCAO	EAP	Rat	1 mA, 15 Hz, 30 min	GV20	Myocardial: A1↑	Wang (2005)^140^

## 3 ANIMAL MODELS FOR BASIC RESEARCH ON CCVD

With the indirect study of animal models, we can consciously change those factors that are impossible or difficult to exclude under natural conditions, so as to observe the experimental results of the model more accurately and carry out comparative studies with human diseases, which is conducive to more convenient and effective understanding of the occurrence and development of human diseases and research on prevention and control measures. Therefore, this paper systematically combs the animal model of EAP prevention and treatment of CCVD, providing a favorable basis for future research.

In this paper, the common model animals used in the basic research of EAP treatment of CCVD include rats, mice and rabbits. Of the 78 articles we included, 30 involved animal models of cardiovascular disease, including myocardial ischemia/reperfusion injury (MIRI) or myocardial ischemia reperfusion (MIR), myocardial ischemia (MI) or acute myocardial ischemia (AMI), cardiopulmonary resuscitation (CPR), simulative global ischemia and reperfusion (SGIR), cardiopulmonary resuscitation (CPR), cardiopulmonary bypass (CPB), sepsis‐induced cardiomyopathy (SICM), and chronic myocardial ischemia (CMI). There were 16 articles on MIRI/MIR model (accounting for ~53%), 8 articles on MI/AMI model (accounting for ~26%), 2 articles on SGIR model (accounting for ~6%), 1 article on CPR model (accounting for ~3%), 1 article on CPB model (accounting for ~3%), 1 article on CMI model (accounting for ~3%), and 1 article on SICM model (accounting for ~3%) (Table 1). Therefore, MIRI and AMI are major models of cardiovascular disease. Myocardial infarction is ischemic necrosis of myocardium. Specifically, on the basis of coronary artery disease, the blood flow of the coronary artery is drastically reduced or interrupted, resulting in severe and lasting acute ischemia of the corresponding myocardium, and eventually ischemic necrosis of the myocardium.^7^ In AMI models, the anterior descending branch of the left coronary artery (LAD) is usually surgically lapped, causing myocardial ischemia and infarction in the innervated area. MIRI refers to the reconstruction of blood flow after myocardial ischemia (myocardial infarction). When blood re‐enters the infarct site, it causes degeneration, destruction, and necrosis of vascular endothelial cells and cardiomyocytes, resulting in decreased myocardial function. The modeling method is to induce acute myocardial ischemia by ligation of the anterior descending branch of the left coronary artery and to induce myocardial cell injury by blood reperfusion in a short time.^8^, ^9^

In 48 articles on animal models of cerebrovascular disease, involving middle cerebral artery occlusion/reperfusion injury (MCAO), cerebral ischemia/reperfusion injury (CI/RI), vascular dementia (VD), and cerebral ischemia injury. Among them, there were 39 articles on MCAO model (accounting for ~81%), 7 articles on CI/RI model (accounting for ~14%), 1 article on VD model (accounting for ~2%), and 1 article on cerebral ischemia injury model (accounting for ~2%) (Table 1). MCAO model is widely recognized internationally as the standard animal model of focal cerebral ischemia, and its pathogenic mechanism is similar to human cerebral ischemia symptoms.^10^ It is caused by a decrease in blood flow to the cerebral cortex by blocking the middle part of the middle cerebral artery, resulting in ischemia and reperfusion injury to the brain. The most commonly used construction method in recent years is to block the blood supply of the left middle cerebral artery with fishing line, thus causing cerebral ischemia.^11^ CI/RI refers to the phenomenon of further aggravation of tissue and functional injury caused by the restoration of blood perfusion after cerebral ischemia, which is the pathophysiological basis of a variety of cerebrovascular diseases.^12^ The middle cerebral artery was blocked for several hours, and then the thread plug was slowly removed under anesthesia in rats to restore the blood flow of the internal carotid artery and middle cerebral artery, and the blood reperfusion was 24 h, namely, the preparation of the CI/RI model was completed.^13^

All in all, MI and MIRI models are classic animal models for basic research on EAP prevention and treatment of cardiovascular diseases, while MCAO and CI/RI are the most commonly used and relatively mature models in cerebrovascular diseases.

## 4 ACUPOINT SELECTION RULE AND ACUPUNCTURE PARAMETERS IN CCVD ANIMAL RESEARCH

**FIGURE 2:**
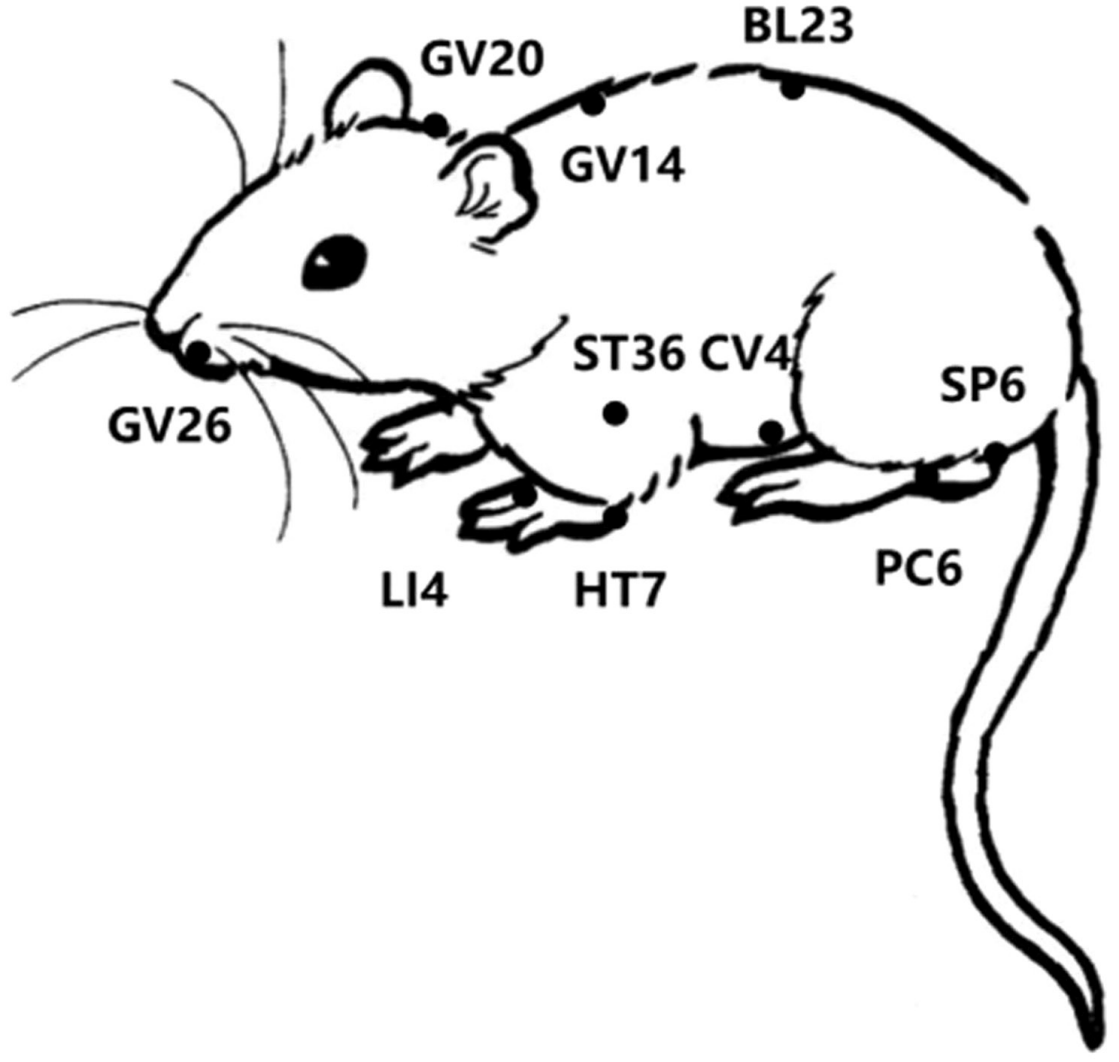
Acupoint compatibility model diagram of EAP for prevention and treatment of CCVD.

The theory of “meridian‐Zang‐fu correlation” is the core content of the meridian theory, which reflects the bidirectional connection between the body surface, meridian‐Zang‐fu.^14^ In the theory of “meridian‐Zang‐fu correlation,” Neiguan (PC6) is one of the acupoints and collaterals of the pericardium meridian, which can treat diseases of the heart and mind, and can harmonize the qi and blood channels, with excellent function, and is commonly used acupoints for the treatment of diseases of the heart and brain system, and the clinical effect is significant.^15^ Among the 30 studies of EAP prevention and treatment of cardiovascular diseases included in this paper, there are 27 studies selected PC6 as EAP acupoints, of which 19 studies used PC6 acupoints alone, and the other 11 reported EAP PC6 acupoints combined with Zusanli (ST36), Guan yuan (CV4), Hegu (LI4), and other acupoints (Table 1). This indicated that pericardial meridians are the main meridians of EAP for prevention and treatment of cardiovascular diseases, and PC6 is the main intervention acupoint (Figure 2). According to the meridians theory of traditional Chinese medicine, Baihui (GV20) acupoint is the meeting of all Yang and the Master of all diseases.^16^ In this paper, among 48 studies on EAP prevention and treatment of cerebrovascular diseases, 42 studies selected GV20 as EAP intervention acupoint, of which 24 studies used GV20 alone, and the other 24 reported that GV20 Sanyinjiao (SP6) or GV20 combined with Dazhui (GV14) were used for EAP combined intervention (Table 1). This shows that the governor vein is the main meridians for the prevention and treatment of cerebrovascular diseases by EAP, and GV20 is the common acupoint (Figure 2).

The current frequency and stimulation intensity of acupuncture intervention are closely related to the therapeutic effect of acupuncture.^6^ Therefore, this paper also combed the acupuncture parameters of EAP for prevention and treatment of CCVD and found that the general duration of EAP treatment was 30 min, the acupuncture depth was mostly distributed between 1.5 and 5 mm, the stimulation intensity was mostly 1 mA, and the low‐frequency (2, 10 Hz), frequency conversion (2/15 Hz, 2/100 Hz), and other stimuli were commonly used (Table 1). The systematic review of this paper is expected to provide reference for the clinical application of EAP in cardiovascular diseases.

To sum up, in cardiovascular disease, the AMI model is commonly used to simulate ischemic injury in the cardiovascular system, while the MIRI model is used to simulate reperfusion injury caused by blood flow recovery after focal tissue ischemia. These are the main models for studying the pathogenesis of cardiovascular diseases. By summarizing the included literature, we found that EAP and similar acupuncture and moxibustion intervention usually use a single point or a combination of multiple points in the selection of points. In cardiovascular disease, PC6 is the most commonly used acupoint. Among other parameters, EAP intervention usually lasts for 30 min, with a needle depth mostly between 1.5 and 5 mm, a stimulation intensity mostly of 1 milliampere, and a commonly used frequency of low frequency. cerebrovascular diseases, the MCAO model is commonly used to simulate ischemic injury in the cardiovascular system, while the CI/RI model is used to simulate reperfusion injury caused by blood flow recovery after focal tissue ischemia. These are the main models for studying the pathogenesis of cerebrovascular diseases. EAP and similar acupuncture and moxibustion intervention usually use a single point or a combination of multiple points. In cerebrovascular diseases, GV20 is the most commonly used.

The main acupoints of EAP for the treatment of cardiovascular diseases are PC6, ST36, CV4, LI4, and HT7. The main acupoints for treating cerebrovascular diseases are GV20, GV14, GV26, BL23, and SP6. The main acupoints for EAP to prevent and treat CCVD were GV20, GV14, BL23, GV26, ST36, CV4, SP6, LI4, HT7, and PC6.

## 5 BIOLOGICAL MECHANISMS OF EAP IN THE PREVENTION AND TREATMENT OF CARDIOVASCULAR AND CEREBROVASCULAR DISEASES

Ischemic tolerance refers to the ability of an organism to acquire temporary resistance to lethal ischemia through several short periods of ischemic preconditioning.^17^ This adaptive response is considered an evolutionarily conserved defense mechanism.^18^ Establishing ischemic tolerance is an effective endogenous protective mechanism to reduce ischemic injury, and mimicking this unique endogenous protective mechanism is a potential strategy to prevent CCVD. EAP is a treatment method that combines traditional Chinese acupuncture and moxibustion and modern science and technology under the thought of treating diseases before they occur. EAP is similar to ischemic pretreatment in that it intervenes before the onset of CCVD and can have a certain therapeutic effect on CCVD. Therefore, the mechanism by which EAP produces this protective effect should be similar to the mechanism by which ischemic pretreatment induces ischemic tolerance. In the past few decades, a significant amount of effort has been devoted to studying the molecular mechanisms of EAP in preventing and treating cardiovascular diseases. The molecular mechanisms involve pathways such as inflammatory response, oxidative stress, autophagy and apoptosis, vascular regeneration, and energy metabolism. Therefore, this article summarizes the current molecular mechanisms of EAP in preventing and treating cardiovascular diseases and discusses the similarities and differences between EAP in preventing and treating cardiovascular diseases and cerebrovascular diseases.

### 5.1 EAP inhibits inflammatory response caused by myocardial ischemic injury

Cardiac cell death caused by acute myocardial infarction (AMI) in cardiovascular disease leads to a series of pathological and physiological reactions in the heart.^19^ This process involves proinflammatory responses and extracellular matrix (ECM) degradation, anti‐inflammatory responses, and the formation of new scars. However, the intense inflammatory response caused by tissue damage leads to excessive cardiac fibrosis, resulting in antagonistic cardiac remodeling, and eventually develops into adverse cardiovascular events such as heart failure.^20^ Immune cells represented by macrophages play an important role in this process.^21^ Ischemia causes myocardial injury, and the damaged myocardium drives circulating monocyte‐macrophages to migrate to the heart and activate monocyte macrophages to polarize into M1 type.^22^ M1 macrophages can clear all dead tissue and necrotic debris through phagocytosis and secretion of proteolytic enzymes.^23^ But usually, the overactivation of M1 macrophages in the infarct area prolongs the inflammatory environment, causing the enlargement of the AMI infarct area, leading to the formation of scar tissue, and simultaneously causing adverse remodeling of the damaged ventricular wall.^24^ Yang et al.^25^ found that EA stimulation of PC6 on mice with AMI can effectively reduce the number of M1 macrophages in the mouse heart, whether as a pretreatment or as an intervention after modeling. It also facilitates the polarization of M1 macrophages to M2 in the hearts of AMI mice. The study^25^ further discovered that after EA intervention, the protein expression levels of inflammatory factors in the myocardial tissue of AMI mice, such as interleukin‐1β (IL‐1β), tumor necrosis factor‐α (TNF‐α), and Toll‐like receptor 4 (TLR4), decreased to varying degrees, with the most significant reduction observed at an electrical stimulation intensity of 1 mA. Consistent with previous results, the impact of an electrical stimulation intensity of 1 mA on the polarization of M1 macrophages to M2 in the cardiac tissue of AMI mice was the most pronounced. This suggests that both acupuncture pretreatment and post‐modeling EA intervention could alleviate inflammation and mitigate myocardial injury caused by AMI by regulating the function and activity of macrophages.

Nuclear factor kappa‐B (NF‐κB), a versatile gene transcription factor, is usually retained in the cytoplasm in an inactive dimeric form (p52/p65 or p50/p65).^26^ The translocation of NF‐κB p65 to the nucleus is a key factor in activating the NF‐κB pathway. When AMI occurs, the damaged myocardial tissue activated of the complement cascade and promoted the production of reactive oxygen species (ROS) and damage‐related molecular patterns, which activated NF‐κB pathway, causing the production of inflammatory factors. Calcitonin gene‐related peptide (CGRP), as one of the most effective anti‐inflammatory neuropeptides,^27^ can reduce the translocation of the NF‐κB complex to the cell nucleus to inhibit its activation.^28^ The release of CGRP mainly depends on the activation of the transient receptor potential vanilloid‐1 (TRPV1) channel.^29^ Wu et al.^30^ found that EAP could downregulate the protein level of NF‐κB p65 in AIM rat myocardial tissue. In addition, after EAP intervention, the expression of TRPV1 and CGRP proteins in AIM rat myocardial tissue significantly increased, indicating that EAP could strengthen the TRPV1/CGRP signal in myocardial tissue, downregulate the expression of NF‐κB p65 protein to alleviate myocardial inflammatory damage, and achieve myocardial protection.

Inflammatory factor IL‐1β is closely related to macrophage activation, and the biological activation of IL‐1β is regulated by the NOD‐like receptor thermal protein domain‐associated protein 3 (NLRP3) inflammasome. The intracellular NLRP3 inflammasome activates caspase‐1 to cleave the inactive precursor pro IL‐1β, activating IL‐1β.^31^ The activity of the NLRP3 inflammasome has a dual signal regulation mode.^32^ The stimulation of the pattern recognition receptor (PRR) of the NF‐κB and mitogen‐activated protein kinase (MAPK) pathways can promote the transcription of genes encoding components of the NLRP3 inflammasome. Zhang et al.^33^ found that in the acute phase of myocardial ischemia, the content of macrophages in the hearts and spleens of AMI mice significantly increased, and NLRP3 and IL‐1β were highly expressed in local myocardial tissue. After EAP at the PC6 acupoint, the expression levels of NLRP3 and IL‐1β in AMI mouse myocardial tissue decreased, and the number of macrophages in the heart and spleen significantly decreased. Splenectomy and EAP could significantly improve the therapeutic effect on AMI mouse heart function.^34^ These results indicate that EAP could activate the TRPV1/CGRP signal in myocardial tissue, downregulate NF‐κB p65, reduce the activity of IL‐1β and NLRP3 inflammasome, thereby alleviating myocardial inflammatory damage and achieving myocardial protection. The effect of EAP preconditioning is not ruled out as being related to regulating the number of splenic macrophages.

### 5.2 EAP alleviated oxidative stress caused by ROS

The dynamic balance of oxygen‐free radicals' production and clearance is disrupted, leading to a large accumulation of active oxygen and triggering oxidative stress, which is a crucial factor leading to the occurrence and development of cardiovascular diseases.^35^, ^36^ Under normal circumstances, mitochondria are the main producers of reactive oxygen species in cells,^37^ mainly regulating the proton gradient in the inner membrane through uncoupling protein 2 (UCP2) to reduce membrane potential and reactive oxygen species.^38^ The imbalance occurs between excessive accumulation of ROS and endogenous antioxidant protection can lead to mitochondrial dysfunction.^39^ Mitochondria are the core source of metabolism and energy production, and the voltage‐dependent anion channel 1 (VDAC1) protein serves as a pore in the outer membrane of mitochondria, providing the main interaction between cellular metabolism and mitochondria.^40^ Previous data shows that injecting functionally normal mitochondria into ischemic myocardial cells in advance can reverse the decline of myocardial cell function and cell apoptosis caused by reperfusion after ischemia, thereby limiting infarct size.^41^ The cardiotoxicity caused by the local anesthetic drug bupivacaine, commonly used in clinical surgeries, is closely associated with mitochondrial damage.^42^ Reports have indicated^43^ that EAP at bilateral PC6, ST36, and ST40 acupoints can alleviate the cardiotoxicity caused by bupivacaine in SD rats. The experiments by Wang et al.^44^ suggest that EAP can mitigate bupivacaine‐induced myocardial injury by regulating mitochondrial function. Bupivacaine can induce mitochondrial dysfunction by downregulating the activity of cytochrome c oxidase (COX) in cardiac myocytes and promoting the production of ROS. EAP not only reverses the inhibition of cytochrome c oxidase (COX) activity caused by bupivacaine but also increases SOD activity, reduces malonic dialdehyde (MDA) content, and enhances the expression of proteins related to mitochondrial function such as UCP2, VDAC1, and B‐cell lymphoma‐2 (Bcl‐2), while downregulating Solute Carrier Family 25 Member 6 (SLC25A6). This maintains the normal function of mitochondria in cardiac myocytes, reduces apoptosis caused by oxidative stress, and consequently alleviates the cardiotoxicity induced by bupivacaine. Therefore, EAP could correct the imbalance between ROS and endogenous antioxidant protection to protect mitochondrial function to alleviate cardiovascular diseases.

### 5.3 EAP could bidirectionally regulate cellular autophagy and inhibited myocardial cell apoptosis

Autophagy is a process of self‐degradation and regeneration within cells, capable of eliminating harmful substances within cells and maintaining cellular stability and health.^45^ Dysregulation of autophagy is closely related to cardiovascular diseases such as myocardial ischemia and heart failure.^46^ Ischemic cells clear damaged organelles within the cell through autophagy, thereby avoiding myocardial hypoxia caused by ROS and ER stress. This process impedes the synthesis of adenosine 5′ ‐triphosphate (ATP) within the cell, which in turn activates adenosine 5′ ‐monophosphate (AMP)‐activated protein kinase (AMPK). AMPK is a key regulator of autophagy in the body during myocardial ischemia, and the activation of AMPK is always accompanied by stimulation of myocardial cell autophagy.^47^ Zeng et al. research^48^ found that EAP on PC6 acupoint in AMI rats could reduce the area of myocardial infarction and induce the formation of AMPK‐dependent autophagosomes. This suggests that EAP may alleviate myocardial infarction injury by promoting the AMPK‐dependent autophagy process. Autophagy‐related liver kinase B1 (LKB1) protein is an upstream kinase of AMPK, mainly distributed in the cell nucleus, playing a role in regulating cellular energy metabolism activities.^49^ When myocardial hypoxia occurs, AMPK can activate phosphofructokinase‐2 (PFK2) in the glycolysis pathway to improve glucose metabolism process.^50^ Wang et al. study^51^ found that EAP could upregulate the expression of LKB1, PFK2, and AMPK in myocardium of AMI rats, indicating that EAP could activate autophagy by upregulating the LKB1/AMPK/PFK2 signaling pathway, improving glucose metabolism, and promoting the survival of damaged myocardial cells. Nutritional deficiencies caused by myocardial cell ischemia can inhibit the activity of the mammalian target of rapamycin (mTORC1).^52^ When AMPK is activated and mTORC1 activity is inhibited, it triggers the activation of ULK1 (unc‐51 like autophagy activating kinase 1) and initiates cellular autophagy. EAP promotes autophagic flux by inhibiting the PI3K‐Akt–mTOR pathway and alleviates myocardial ischemia–reperfusion injury (MIRI) damage.^53^ Therefore, autophagy primarily plays a protective role for myocardial cells during the phase of ischemia.^54^

However, during the reperfusion phase, autophagy has the opposite effect. Unlike the ischemic phase, excessive autophagy degrades normally functioning organelles and proteins, eventually leading to autophagic cell death and secondary damage to cells and tissues.^54^, ^55^ In the reperfusion phase, the restoration of blood flow leads to an overproduction of reactive ROS, which upregulates recombinant Beclin1 (Beclin‐1), which is the primary positive regulator of autophagy during the myocardial reperfusion phase.^56^ The interaction of Beclin‐1 on the endoplasmic reticulum phagosome membrane with the autophagy related 14 (Atg14) complex can induce the formation of the autophagosome's double‐membrane structure, which is the initial and crucial stage of autophagy formation.^57^ Studies have found that EAP could inhibit excessive autophagy to prevent tissue damage caused. Research by Chen et al. found^58^ that EAP at acupoint PC6 for different durations can alleviate symptoms of myocardial ischemia–reperfusion injury in AIM rats. Among them, the intervention time of 4–5 days had the best effect, and the myocardial infarction area value decreased after EAP. The expression of ULK1 and Beclin‐1 proteins in rat myocardial tissue was downregulated, indicating that EAP can inhibit excessive cellular autophagy by downregulating the expression levels of ULK1 and Beclin‐1 proteins, reducing myocardial ischemia–reperfusion injury plays a protective role in the myocardium.

Prolonged ischemia in tissues will inevitably lead to cell apoptosis.^59^, ^60^, ^61^ Evidence suggests that EAP could play a crucial role in myocardial protection by inhibiting cell apoptosis. Liu et al.'s experiment^62^ showed that EAP could reduce myocardial cell apoptosis by inhibiting Farnesoid X receptor/Small heterodimer partner (FXR/SHP), downregulating the transcription level of apoptosis inducing factor (AIF), and promoting the expression of Heat Shock Protein 70 (HSP70). Both FXR and SHP belong to the nuclear receptor superfamily and are regulators of cell apoptosis,^63^ while AIF is a direct effector of cell apoptosis,^64^ and HSP70 can block the nuclear translocation of apoptosis inducing factor AIF in mitochondria.^65^ So, EAP could inhibit myocardial cell apoptosis by inhibiting FXR/SHP/AIF and promoting HSP70 to protect against myocardial injure.

### 5.4 EAP inhibited the overload of Ca^2+^ of myocardial cell

Ca^2+^ overload is one of the crucial mechanisms of cardiac injury induced by reperfusion after ischemia. EAP could alleviate cardiac injury through inhibiting overload of Ca^2+^ in myocardial cells. β‐adrenoceptors (β‐AR) play a crucial role in promoting myocardial ischemic injury.^66^, ^67^ Several experiments suggest that the mechanism of EAP's protective effect on the heart of MIRI rats through the PC6 acupoint is related to β‐AR.^68^, ^69^, ^70^, ^71^, ^72^ Cyclic adenosine monophosphate (cAMP) and stimulatied adenylate cyclase g protein (Gs) are important components of the β1‐AR signaling pathway.^73^ Their elevated expression could lead to overload of Ca^2+^ in myocardial cells.^74^ While cardiac arrhythmias induced by reperfusion after myocardial ischemia are related to the loss of Connexin43 (Cx43) in myocardial cells.^75^ Studies have found that EAP at the PC6 acupoint could alleviate MIRI‐induced myocardial infarction by significantly inhibiting Ca^2+^ overload or oscillation, through reducing the expression of β1‐AR, cAMP, Gs protein, and inhibit the loss of Cx43 in myocardial cells of MIRI.^69^, ^76^, ^77^, ^78^ Therefore, EAP inhibited Ca^2+^ overload or oscillation, through reducing the expression of β1‐AR, cAMP, Gs protein, and inhibit the loss of Cx43 in myocardial cells of MIRI to alleviate MIRI‐induced myocardial infarction.

### 5.5 EAP decreased the accumulation of glutamic by ischemia

In cardiovascular diseases, transient forebrain or global ischemic injury induced by cardiac arrest, or cardiac surgery, can lead to the accumulation of glutamic (Glu).^79^ The expression of glutamate receptors is highly abundant in myocardial cells, and excessive accumulation of Glu can continuously stimulate glutamate receptors, ultimately leading to myocardial or neuronal cell damage.^80^, ^81^ The secretion of Glu is mainly related to glutamatergic neurons, which are highly distributed in fastigial nucleus (FN).^82^ FN is the oldest nucleus in the cerebellar developmental system, which is associated with neuroprotection and reflex vasoconstriction.^83^ Previous studies have shown that FN is related to various non motor structures and actively participates in the regulation of visceral activities such as cardiovascular, respiratory, and feeding.^84^ Zhou et al.^85^ found that EAP of HT7 in MIRI model rats can improve sympathetic parasympathetic balance and alleviate MIRI‐induced cardiac damage. Compared with MIRI model rats without EAP, the results showed that EAP inhibited the frequency of sympathetic discharge and the activation of glutamatergic neurons in FN. And by directly inhibiting the glutamatergic neurons in FN, the protective effect of EAP can be replicated. Activation of glutamatergic neurons in FN followed by EAP will eliminate the protective effect. Meanwhile, Yu et al.^86^ found that EAP could alleviate myocardial cells ST‐segment elevation and arrhythmia scores in MIRII model rats by promoting γ‐aminobutyric acid(GABA) levels in the lateral hypothalamus (LHA) and FN and reducing Glu release. When γ‐aminobutyric acid type A (GABAA) receptors are activated, it will lead to sympathetic nervous excitement, which further exacerbates myocardial cell damage,^87^ while EAP could alleviate myocardial injury in MIRI rats by inhibiting the specific activation of GABAA receptors of FN and inhibiting sympathetic nervous excitement.^81^ Therefore, EAP alleviates myocardial injury by reducing the accumulation of Glu, which is closely related to the inhibition of sympathetic nervous system excitation. Recently, new reports^88^, ^89^ have shown that EAP can also improve MIRI by inhibiting sympathetic nervous system by suppressing corticotropin‐releasing hormone neurons in the paraventricular nucleus of the hypothalamus. The above results indicate that inhibiting excessive excitation of the sympathetic nervous system is an important mechanism for EAP to prevent and treat MIRI, and reducing the accumulation of Glu is an important pathway in this regard.

### 5.6 EAP promoted vascular regeneration and protected vascular homeostasis

It is well known that myocardial cell injury stimulates the proliferation and migration of endothelial cells and the formation of new vascular structures as a protective mechanism.^90^, ^91^ This mechanism can alleviate myocardial injury by enhancing angiogenesis and maintaining vascular integrity, thereby increasing cardiac blood flow and promoting the delivery of essential nutrients and oxygen, as well as improving the clearance of metabolic waste.^92^ Hypoxia‐inducible factor‐1α (HIF‐1α) and vascular endothelial growth factor (VEGF) play an important role in promoting angiogenesis. Fu et al.^93^ found that EAP at PC6, CV14, and ST36 acupoints could effectively reduce infarction volume and improve heart function in rats with chronic myocardial ischemia. At the same time, it was found that the expression of HIF‐1α and VEGF in rat serum and myocardial area was upregulated after EAP.^93^ These results suggest that EAP could promote angiogenesis by promoting the expression of HIF‐1α and VEGF in the myocardial area, to effectively prevent and treat chronic myocardial ischemia.

Endothelin (ET) is an important factor in regulating cardiovascular function, and plays an important role in maintaining the basal vascular tension and the homeostasis of the cardiovascular system. Myocardial hypoxia can cause a large release of ET, leading to myocardial ischemic necrosis, and exacerbating myocardial ischemia–reperfusion injury.^94^ Wang et al.^95^ found that EAP and moxibustion pretreatment can significantly reduce plasma ET and serum creatine kinase (CK) levels and promote the expression of HSP70, and this preventive protective effect still exists within 48 hours. These results suggest that needle and moxibustion pretreatment has a preventive protective effect on MIRI, related to the reduction of plasma ET levels, serum CK levels, and the enhancement of myocardial tissue HSP70 expression, and this preventive protective effect also has a delayed protective effect.

**FIGURE 3:**
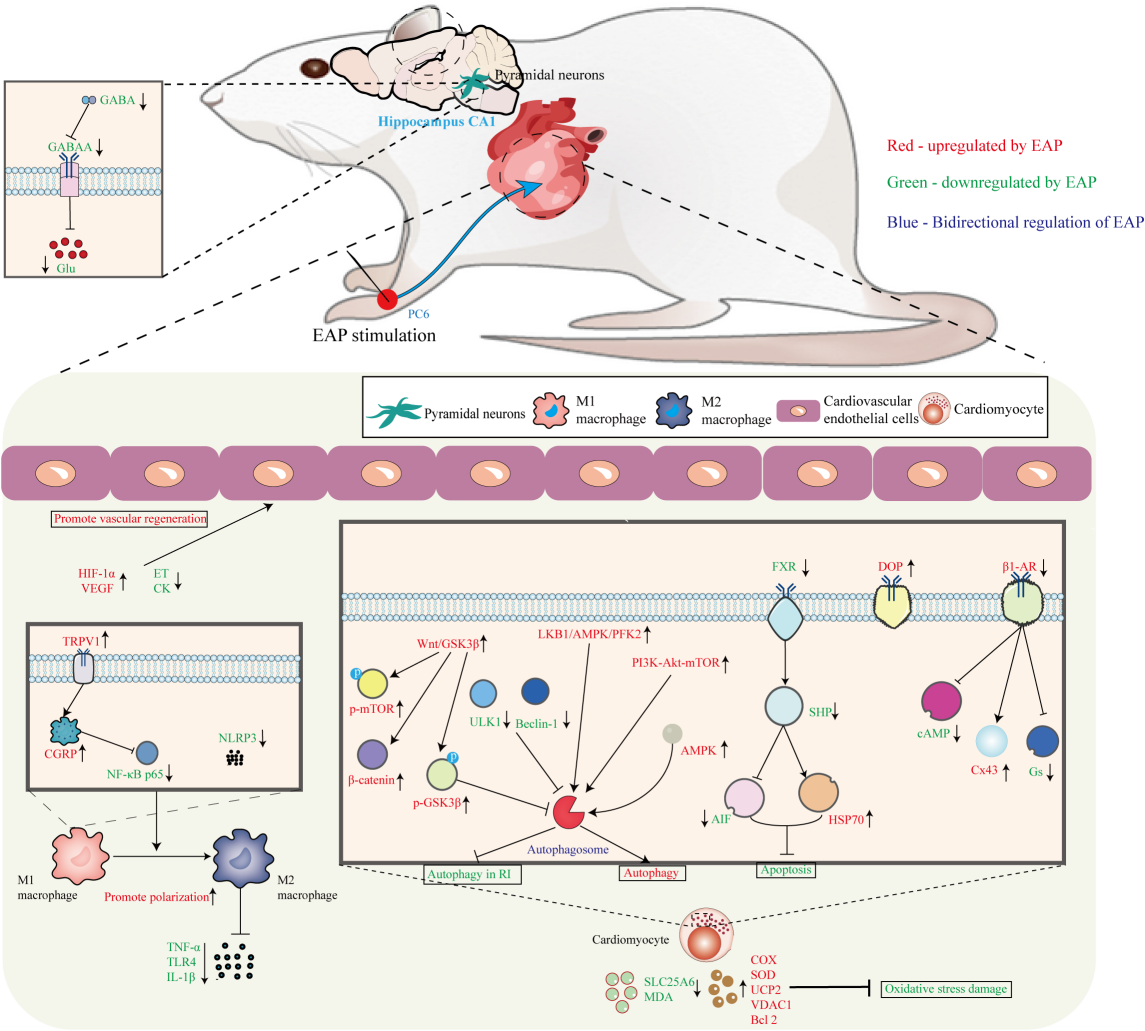
Biological mechanisms of EAP in prevention and treatment of cardiovascular diseases.

To sum up, the mechanisms of EAP in preventing and treating cardiovascular diseases mainly involve pathways such as inflammatory response, oxidative stress, apoptosis and autophagy, calcium overload, glutamate metabolism, and vascular regeneration (Figure 3). EAP has clear anti‐inflammatory effects in cardiovascular diseases, mainly involving macrophage polarization and activation of the TRPV1/CGRP signaling pathway. The antioxidant effect of EAP is mainly related to reducing mitochondrial damage during oxidative stress. During myocardial ischemia, EAP can inhibit myocardial cell apoptosis and promote the formation of autophagosomes, mainly involving the FXR/SHP, LKB1/AMPK/PFK2, and PI3K Akt mTOR signaling pathways. During the reperfusion phase of blood circulation recovery, EAP can inhibit the formation of excessive autophagy, which is related to the activation of the Wnt/GSK3 β signaling pathway. Finally, EAP can inhibit Ca^2^ + overload or oscillation by reducing the expression of β 1‐AR, cAMP, and Gs proteins, promote Glu metabolism, reduce myocardial damage caused by sympathetic nervous system excitation, and enhance the expression of HIF‐1 α and VEGF in the myocardial area, promote angiogenesis, and improve heart function.

The figure shows the molecular mechanisms involved in the prevention and treatment of cardiovascular disease by EAP (red represents upregulation, green represents downregulation, and blue represents bidirectional regulation). EAP can promote macrophage polarization toward the M2 type, reducing the expression of IL‐1β, TNF‐α, Toll‐like receptor TLR4, and NLRP3 in myocardial tissues, as well as activating the TRPV1/CGRP signaling pathway to downregulate NF‐κB p65, thus exerting an anti‐inflammatory effect. During the myocardial ischemic phase, it enhances the activity of COX and SOD, upregulates the expression of UCP2, VDAC1, and Bcl‐2 proteins, or downregulates the levels of SLC25A6 and MDA, alleviating ROS‐induced oxidative stress. Inhibition of the FXR/SHP signaling pathway downregulates the transcription level of AIF, and the promotion of HSP70 expression inhibits myocardial cell apoptosis. Autophagy formation is encouraged by inducing the formation of AMPK‐dependent autophagosomes and activating the LKB1/AMPK/PFK2 or PI3K‐Akt‐mTOR signaling pathways. In the reperfusion phase, excessive autophagy is inhibited by downregulating ULK1 and Beclin‐1 or activating the Wnt/GSK3β signaling pathway to upregulate the protein levels of p‐mTOR, p‐GSK3β, and β‐catenin. Myocardial injury is mitigated by increasing the expression of δ‐opioid receptors in the myocardium, inhibiting the loss of Cx43 in myocardial cells, and suppressing the activation of GABAA receptors in FN, thus reducing sympathetic nervous excitement. Cardiac calcium overload is inhibited by reducing the expression of β1‐AR, cAMP, and Gs protein. Excessive accumulation of Glu is decreased by elevating the levels of GABA in the LHA and FN. Cardiac vascular regeneration is promoted by enhancing the expression of HIF‐1α and VEGF in the myocardial region, or by reducing the content of plasma ET and serum CK in the myocardium, as well as by enhancing the expression of HSP70.

## 6 BIOLOGICAL MECHANISMS OF EAP IN THE PREVENTION AND TREATMENT OF CEREBROVASCULAR DISEASES

### 6.1 The anti‐inflammatory effects of EAP in cerebrovascular diseases

In cerebrovascular diseases, similar to cardiovascular diseases, macrophages also play a vital role. Microglial cells—the macrophages of the central nervous system—are widely distributed in the brain. After ischemic stroke, trauma, or other neurological injuries, these resting microglial cells can be activated and polarized into M1‐type microglia, leading to neuroinflammation.^96^ The transformation of microglia into the M2 phenotype is considered a potential therapeutic target for suppressing neuroinflammation and providing neuroprotection.^97^ Li et al.^98^ found that percutaneous acupoint electrical stimulation (TEAS) preconditioning at GV20, SP6, and bilateral ST36 could improve CI/RI following MCAO and suppress the onset of neuro‐inflammation. Further, TEAS preconditioning can inhibit the activation state of microglia after CI/RI and promote the polarization of microglia from M1 to M2 phenotype. Apart from this, the study also discovered that TEAS could stimulate the expression of NF‐E2‐related factor (Nrf2) and Heme oxygenase‐1 (HO‐1) proteins.^99^ The Nrf2/HO‐1 pathway is known to be a significant signaling pathway in the body's oxidative stress response.^100^ Numerous studies^101^, ^102^ have demonstrated that activating the Nrf2/HO‐1 pathway can alleviate damage caused by ischemic reperfusion. These results suggest that TEAS preconditioning can improve a rat's CI/RI by inducing the polarization of microglia to M2 type, suppressing neuroinflammation, and this process might be associated with the Nrf2/HO‐1 pathway. The Annexin A1 (ANXA1) and formyl peptide receptor (FPR) pathways have been shown to exert anti‐inflammatory, analgesic, and other protective effects in various organs and systems in the human body.^103^ Zou et al.^104^ found that EAP CV4 and Changqiang (GV1) acupoints enhanced the expression of ANXA1 and FPR, inhibited the activation of M1‐type microglia induced by MCAO, reduced the release of pro‐inflammatory cytokines like IL‐1β, inducible isoform of nitric oxide synthase (INOS), TNF‐α, and increased the release of anti‐inflammatory cytokines like arginase‐1 (Arg‐1), and brain‐derived neurotrophic factor (BDNF), improving neurological clinical performance, learning, and memory impairments in MCAO mice, and reduced the infarct volume. These results suggest that EAP regulation of microglial polarization towards M2 may be related to ANXA1/FPR axis.

The ischemic penumbra, severely ischemic but salvageable tissue, is triggered by ischemia.^105^ After ischemic brain injury, numerous cellular effects occur in the ischemic penumbra, such as a protective increase in Yes‐associated protein (YAP) expression in the cortical ischemic penumbra.^106^ It has been reported^107^ that EAP promotes YAP expression in the cortical ischemic penumbra of MCAO model rats, decreasing the levels of proinflammatory factors such as IL‐1β, IL‐6, and TNF‐α in this region as YAP levels rise. However, the specific mechanism between YAP upregulation and the reduction of the inflammatory response is not yet clear.

Within a few hours of cerebral ischemia, an intense inflammatory response is triggered, and the High mobility group box 1 protein (HMGB1) is released from early damaged neurons, acting as a mediator connecting acute brain injury and subsequent inflammatory processes.^108^, ^109^ Endogenous anti‐inflammatory mechanisms can prevent damage caused by excessive immune responses, and the cholinergic anti‐inflammatory pathway is a physiological mechanism by which the nervous system interacts with the innate immune system to suppress systemic inflammatory responses.^110^ There is evidence that the α7 nicotinic acetylcholine receptor (α7nAChR) is crucial in mediating cholinergic anti‐inflammatory signal transduction,^111^ and the α7nAChR‐dependent cholinergic signal is related to the release of HMGB1.^112^ Wang et al.^113^ found in their experiments that the expression of α7nAChR significantly decreased after reperfusion in MCAO model rats, at the same time the levels of HMGB1 in the brain and plasma increased. However, these results^113^ were reversed after EAP was performed. Moreover, the effects produced after preconditioning with an α7nAChR agonist were similar to those of EAP, while an α7nAChR antagonist antagonized the effects of EA. These results suggest that EAP can provide robust protection for brief cerebral ischemic injury in rats by activating α7nAChR to inhibit the release of HMGB1. Simultaneously, the inhibition of HMGB1 release can alleviate the apoptosis of brain neurons.

### 6.2 EAP‐alleviated oxidative stress caused by ROS in cerebrovascular diseases

Similar to cardiovascular disease, EAP can exert antioxidant stress by reducing ROS production and enhancing endogenous antioxidant protective factors in cerebrovascular diseases. Nicotinamide adenine dinucleotide phosphate (NADPH) oxidase, a multi‐subunit membrane‐associated protein, is the primary initiator of ROS generation.^114^ Guo et al.^115^ showed that EAP reversed the increased expression of NADPH oxidase in the MCAO group. Endothelial nitric oxide synthase (eNOS) and its product NO play a crucial role in neuroprotection.^68^ The lack of eNOS can promote inflammation and cell death in brains of mice caused by cardiac arrest/cardiopulmonary resuscitation (CA/CPR). Yong et al.^116^ found that EAP at the GV20 acupoint in mice could protect against brain injury after CA/CPR by reducing oxidative stress and neuroinflammatory factors, inhibiting neuronal apoptosis, and increasing the production of eNOS.^117^ There have also been reports^118^ stating that electroacupuncture can increase the activity of antioxidant enzymes such as thioredoxin (Trx), SOD, and glutathione peroxidase (GSH‐Px), inhibit the expression of MDA in brain ischemic injury, thereby producing a protective effect against oxidative stress. Some articles report that after cerebral ischemia, TRPV‐1‐KO mice have lower neurological and motor deficits and infarct volumes than normal wild‐type mice. Ischemic stroke can activate the TRPV‐1 channel in the brain.^119^ Long et al.^120^ found that EAP at the GV20, bilateral BL23, and SP6 acupoints in rats with a MCAO model could reduce mitochondrial damage caused by ischemic reperfusion injury after MCAO by inhibiting the activation of TRPV‐1, thereby exerting a protective effect against oxidative stress. These studies show that EAP could protect mitochondrial function by inhibiting the activation of the TRPV‐1 signaling pathway, upregulating the expression of antioxidant enzymes such as eNOS, Trx, SOD, and GSH‐Px, and downregulating the expression of the main ROS‐generating molecule, NADPH oxidase and MDA, to reduce neuronal injury caused by oxidative stress.

### 6.3 EAP could bidirectionally regulate cellular autophagy and inhibit cell apoptosis in cerebrovascular diseases

During brain tissue ischemia, due to the limited ability of cells to differentiate and regenerate, on the one hand, ischemic cells can degrade redundant, misfolded proteins inside the cell through autophagy, providing energy for the cell, promoting material circulation and cell self‐renewal.^121^ This process is related to the elimination of mitochondria mediated by mitosis, and the overexpression of triggering receptor expressed on myeloid cells‐2 (TREM2) can promote this process.^122^ For example, in TREM2‐deficient Alzheimer's disease mice, the number of mitochondria and ATP levels are reduced compared to normal Alzheimer's disease mice, and the important regulating factor of autophagy, Mammalian target of rapamycin (mTOR) also has functional defects.^123^ Therefore, neuronal TREM2 may be a potential therapeutic target for cerebral ischemia. Yang et al. found^124^ that EAP could upregulate the expression of TREM2 on excitatory neurons in MCAO model mice, and after EAP, autophagy increases, and brain tissue damage caused by ischemia is also reduced. However, the neuroprotective effect of EAP on cerebral ischemia disappears after neuronal TREM2 is knocked out. This suggests that EAP inhibits brain tissue damage by promoting the autophagy process, which is dependent on TREM2.

Similar to EAP in the treatment of cardiovascular disease, EAP can also inhibit excessive autophagy caused by ischemia reperfusion in cerebrovascular disease. During the reperfusion phase, the restoration of blood flow leads to an overproduction of reactive ROS, which promotes the expression of autophagy‐related protein Microtubule‐Associated Protein 1 Light Chain 3 (LC3) and the formation of autophagosomes.^125^ It has been established that LC3 exists in two forms: LC3‐I and LC3‐II, with LC3‐II being enriched on the vesicle membrane, and promoting the formation of mature autophagosomes.^126^ Multiple experiments^127^, ^128^, ^129^, ^130^ also show that EAP's inhibitory effect on autophagy during the reperfusion stage was also related to LC3‐I, LC3‐II, and Beclin‐1 in cerebrovascular disease. Multiple experiments have indicated that electroacupuncture protection (EAP) inhibits autophagy during the reperfusion phase, which is associated with autophagy‐related proteins such as LC3‐I, LC3‐II, and Beclin‐1. For example, EAP can downregulate the expression of autophagy‐related proteins including LC3‐I, LC3‐II, and Beclin‐1. EAP is able to activate the mTOR signaling pathway, leading to the downregulation of p‐ULK1 and FUN14 domain containing 1 (FUNDC1) expression and activate the Wnt/GSK3β signaling pathway, resulting in the upregulation of protein levels of p‐mTOR, p‐GSK3β, and β‐catenin. Additionally, EAP can activate the SIRT1‐FOXO1 signaling pathway, reducing the expression of LC3 in cortical neurons and the ratio of LC3‐II to LC3‐I, and promoting the expression of the p62 protein, thereby inhibiting the formation of autophagy. Further studies^131^, ^132^ have found that both EAP and moxibustion pretreatment can alleviate neuronal apoptosis caused by excessive autophagy following reperfusion by downregulating LC3‐I, LC3‐II, Beclin‐1, and the LC3‐II to LC3‐I ratio. Notably, although both treatments can yield similar protective effects, EAP is generally more effective. These results suggest that during the reperfusion phase, EAP mitigates neuronal damage caused by excessive autophagy by downregulating autophagy‐related proteins such as LC3‐I, LC3‐II, and Beclin‐1, activating mTORC1 signaling to downregulate p‐ULK1 and FUNDC1, or by activating the SIRT1‐FOXO1 signaling pathway. This results in a reduction of LC3 expression in the cortical neurons, a decrease in the ratio of LC3‐II to LC3‐I, and an increase in the expression of the p62 protein, thereby alleviating damage from excessive autophagy.

There is also evidence that EAP could also play a crucial role in neuroprotection by inhibiting cell apoptosis. For instance, EAP could reduce the expression of TRPV1 in MCAO rats, inhibiting NF‐κB transcriptional activity, exerting an anti‐apoptotic effect, and alleviating neurological injury after reperfusion.^133^ Glucose regulated protein 78 (GRP 78) serves as a chaperone protein to maintain endoplasmic reticulum homeostasis in the ischemic brain area, playing an antiapoptotic role,^134^ while growth arrest and DNA damage‐inducible gene 153 (GADD 153) is an important signaling molecule transitioning from anti‐apoptosis to pro‐apoptosis.^135^ Cheng et al.^136^ found that EAP could upregulate the expression of GRP 78 and downregulate the expression of GADD 153 of the ischemic brain area, along with effectively inhibiting the number of apoptotic neurons and improving neuronal survival rate in the CA1 of CI/RI rats. This indicates that EAP could alleviate ischemia‐induced brain cell apoptosis by inhibiting the TRPV1/NF‐κB signaling pathway or upregulating GRP 78 protein and downregulating GADD 153 protein, in cerebrovascular diseases.

### 6.4 EAP alleviated CA1 neurons injury through inhibiting overload of Ca^2+^

EAP also could alleviate CA1 neurons injury through inhibiting overload of Ca^2+^. The α‐amino‐3‐hydroxy‐5‐methyl‐4‐isoxazole‐propionic acid receptor (AMPA) type glutamate receptor (AMPAR) was a heteromeric complex composed of Glutamate receptor 1–4 (GluR1‐GluR4).^137^ Cerebral ischemia could induce a downregulation of GluR1 protein, leading to the AMPAR‐mediated excessive Ca^2+^ influx of CA1 pyramidal neurons.^138^ GluR1 has been reported to mediate the protective effect of EAP on CA1 neurons. Liu et al.^139^ found that EAP treated with GV20 could reduce the death of CA1 pyramidal neurons in BCCAO mice and was associated with significantly increased expression of GluR1 protein. But in GluR1 protein knockout BCCAO mice, the effect of EAP on protecting CA1 pyramidal neuron damage was disappeared.^139^

In some research reports suggest that endogenous cannabinoid system also were involved in the regulation of GluR1 of EAP.^140^, ^141^, ^142^, ^143^, ^144^ Wang et al.^141^ showed that both the area of cerebral infarction and neuronal damage were alleviated by EAP, and this effect might be related to the increased of endogenous cannabinoid 2‐arachidonoyl glycerol (2‐AG). He et al.^145^ showed in their experiments that EAP can increase the number of pyramidal neurons in the CA1 area of the hippocampus after reperfusion in the MCAO model. This effect of EAP is related to the upregulation of the Wnt/β‐catenin signaling pathway; Wnt/β‐catenin has been proven to have a protective effect in lethal tissue ischemia.^146^ Moreover, the early use of Wnt/β‐catenin agonist LiCl can produce the same therapeutic effect as EAP, but unlike EA treatment, this effect requires long‐term application of LiCl.^147^ Notably, the experimental results^139^ of cannabinoid subtype 1 receptor (CB1R) agonists and antagonists showed that EAP reducing the amount of Ca^2+^ influx in CA1 pyramidal neurons by upregulating the expression of GluR1 protein was dependent on the activation of CB1R. Subsequently, Yang et al.^148^ demonstrated the activation effect of EAP on the endogenous cannabinoid system, and also EAP‐induced ischemic tolerance is related to the activation of CB1R in astrocytes. Therefore, EAP protected CA1 neurons may by activating the cannabinoid system to upregulate GluR1 and prevent excessive Ca^2+^ influx.

### 6.5 EAP improved brain injury by protecting the blood–brain barrier

The integrity of the blood–brain barrier (BBB) is crucial for maintaining appropriate neural function and protecting the central nervous system from injury and disease.^149^, ^150^ Ischemic injury and CI/RI caused by thrombolytic treatment can lead to the breakdown of the integrity of the BBB.^151^ Jung et al.'s^152^ results show that EAP could improve neurological function after ischemic injury by reducing the production of ROS and the expression of NADPH oxidase 4 (NOX4), alleviating the severity of brain edema, which may be related to the recovery of BBB function. Tight junctions (TJ) are an important structural component of the BBB, which seal the gaps between adjacent endothelial cells.^153^ Claudin and occludin are key transmembrane proteins that form these seals.^154^ In the BBB integrity damage caused by ischemic brain tissue damage, there is a phenomenon of a large amount of loss of TJ proteins.^155^, ^156^ Zou et al.^157^ found that EAP GV20 could significantly reduce the permeability of the BBB and also improved the symptoms of brain edema in MCAO model rats, which accompanied by significantly reduce the loss of TJ proteins (including claudin‐5 and occludin) in the brains. In the formation of new blood vessels, VEGF and matrix metalloproteinase‐9 (MMP‐9) will induce the dissolution of the ECM, providing the necessary conditions for the formation of new blood vessels. This process will destroy the integrity of the tight junctions of endothelial cells and simultaneously cause an increase in BBB permeability.^158^ Lin et al.^159^ found that EAP could downregulate the proportion of MMP‐9‐positive cells in the BBB tissue of CI/RI rats, and downregulate the transcription levels of MMP‐9 and VEGF in rat brains. And the inhibitory effect produced by EAP at different times is different, with EAP for 15 days being the most effective. These results suggest that EAP protected the BBB in two ways: first, it protected the BBB by reducing the flow of TJ protein; second, by reducing the expression of MMP‐9 and VEGF to reduce ECM degradation, thereby reducing the destruction of the BBB caused by new angiogenesis.

### 6.6 EAP promoted vascular regeneration

EPH receptor B4 (EphB4) and its ligand Ephrin B2 (EphrinB2) play a crucial role in vascular development.^160^ Nonreceptor tyrosine kinase (Src) and PhosphatidylINOSitide 3‐kinases (PI3K) are the hubs in the two signal transduction pathways mediated by EphB4/EphrinB2. Wu et al.^161^ found that EAP can enhance the transcription levels of EphB4 and EphrinB2 in the brains of MCAO model rats, promoting angiogenesis. And it was also found that EAP could significantly increase the expression levels of Src and PI3K, and the effect of EAP promoting angiogenesis disappeared after using Src/PI3K inhibitors.^162^ These results suggest that EAP could promote angiogenesis and which may be related to the Src/PI3K/EphrinB2/EphB4 signaling pathway.

### 6.7 EAP enhances nerve growth factor and activates neurotransmitter receptors

BDNF is a member of the neurotrophic factor family and plays an important role in neural plasticity, neuron development, differentiation, and survival.^163^, ^164^ Evidence has shown that both exogenous and endogenous BDNF can promote the plasticity of synapses and axonal growth, which is positively correlated with behavior changes and neurological recovery caused by CI/RI injury.^165^, ^166^, ^167^ Research has found that repeated stimulation of GV20 and GV14 with EAP in a mild focal brain ischemia model can alleviate reperfusion‐induced brain injury by promoting the expression of BDNF.^168^ Kim et al.'s^169^ study showed that EAP at GV20 and GV14 acupoints could induce ischemic tolerance in mice by promoting the expression of BDNF. Except for BDNF, there were also reports that EAP could increase the expression of neurotrophic factors such as insulin‐like growth factor 1, basic fibroblast growth factor, and glial cell‐derived neurotrophic factor.^170^, ^171^ So, EAP could improve brain nerve function by enhancing the expression of BDNF.

Previous studies have found that EAP can activate CB1R, and the activation of CB1R by EAP will cause changes in the expression of various protein molecules.^172^, ^173^, ^174^, ^175^, ^176^, ^177^ For example, in the rat MCAO model, EAP can promote the expression of extracellular‐regulated protein kinases 1/2 (ERK1/2), epsilon protein kinase C (εPKC), signal transducers and activators of transduction‐3 (STAT3), and the phosphorylation of Glycogen synthase kinase3β (GSK‐3β) by activating CB1R. The promotion of GSK‐3β phosphorylation by EAP can not only be achieved through CB1R, but EAP can also increase the phosphorylation level of GSK‐3β by activating adenosine A1 receptor (ADORA1) and adiponectin receptor agonist 1 (AdipoR1).^178^, ^179^ In cerebrovascular diseases, the appropriate activation of ERK1/2 enhances neuronal survival and regeneration,^180^ STAT3 activation promotes angiogenesis,^181^ εPKC activation mitigates post‐ischemic mitochondrial dysfunction to confer neuroprotection,^182^ and increased phosphorylation of GSK3β can ameliorate neurological deficits and cognitive impairments.^183^ In addition, other study^184^ has found that repeated EAP stimulation of the GV20 acupoint can increase the expression of enkephalin in the brain and activate delta opioid receptors and μ‐opioid receptors, and this process can have a protective effect on ischemic brain tissue. Finally, there are reports^185^ saying that the Notch signal is also involved in the focal cerebral ischemic tolerance induced by EAP. However, whether activating the Notch signal is beneficial to ischemic brain tissue remains controversial.^186^

**FIGURE 4:**
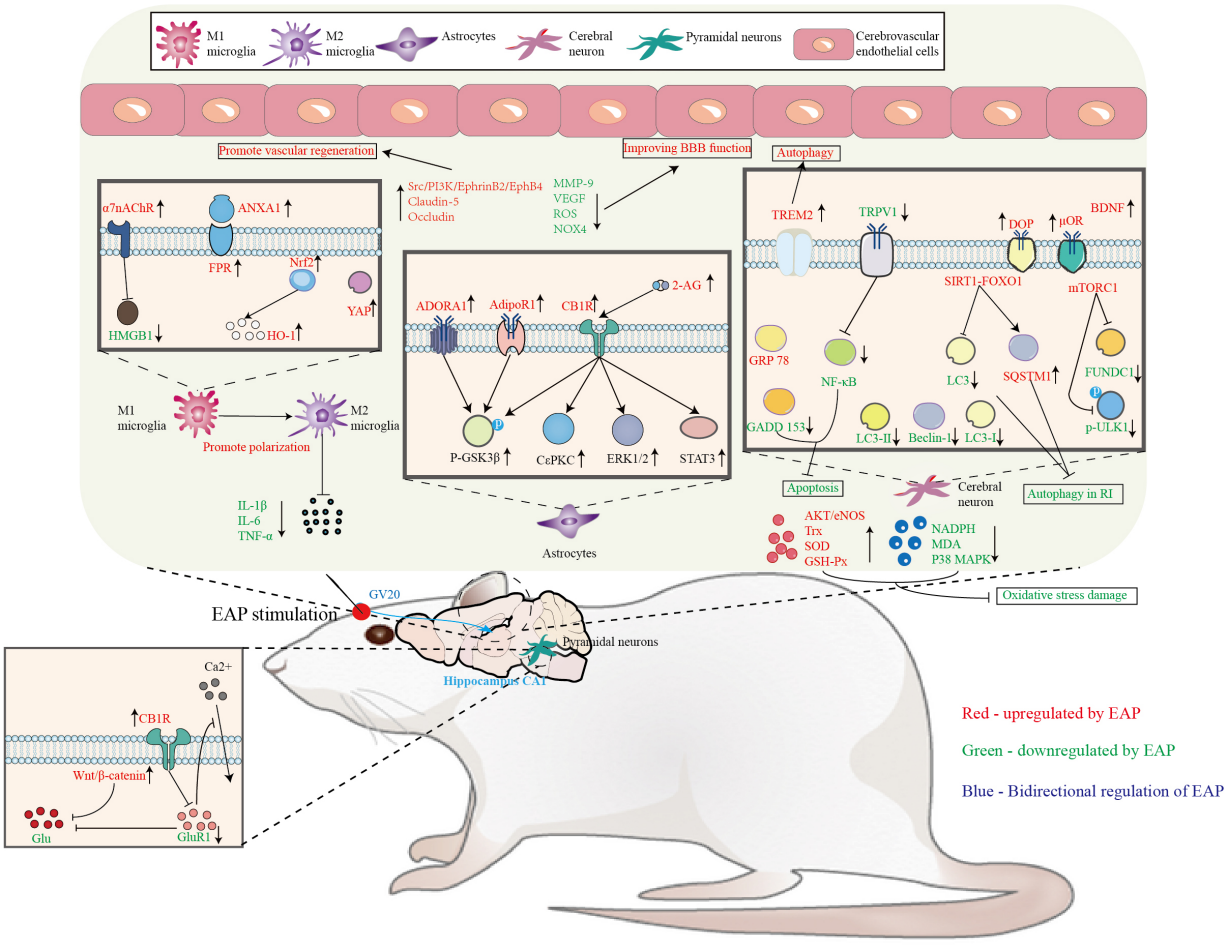
Biological mechanisms of EAP in prevention and treatment of cerebrovascular diseases.

To sum up, the molecular mechanism and prevention of EAP in the prevention and treatment of cerebrovascular diseases mainly involve pathways such as inflammatory response, oxidative stress, apoptosis and autophagy, calcium overload, blood–brain barrier homeostasis, and secretion of neurotrophic factors and neurotransmitters (Figure 4). In cerebrovascular diseases, promoting the polarization of microglia toward M2 type may be a key mechanism for the anti‐inflammatory effect of EAP, and activating the Nrf2/HO‐1 and ANXA1/FPR signaling pathways is an important pathway for producing anti‐inflammatory effects. In antioxidant stress, EAP can activate the AKT/eNOS signaling pathway and downregulate the expression of the main molecules involved in ROS production, NADPH oxidase, and MDA, to reduce nerve damage caused by oxidative stress. During cerebral ischemia, EAP can inhibit brain cell apoptosis by inhibiting the TRPV1/NF‐κB signaling pathway and enhance autophagy formation by upregulating the expression of TREM2 in brain neurons. During the reperfusion phase, EAP inhibits excessive autophagy by activating the mTORC1\p‐ULK1 and SIRT1‐FOXO1 signaling pathways and promoting the expression of p62 protein. Finally, EAP can promote the expression of GluR1 and reduce Ca^2+^ influx by activating the CB1R receptor, activate the Src/PI3K/EphrinB2/EphB4 signaling pathway to promote vascular regeneration and improve blood–brain barrier function, and enhance the expression of BDNF, activate the expression of δ‐opioid receptors and μ‐opioid receptors to improve brain nerve function.

The figure shows the molecular mechanisms involved in the prevention and treatment of cerebrovascular diseases by EAP (red represents upregulation, green represents downregulation, and blue represents bidirectional regulation). EAP can reduce the expression of proinflammatory cytokines IL‐1 β, IL‐6, and TNF‐α by promoting the expression of YAP, activate α 7nAChR to inhibit the release of HMGB1, and activate Nrf1/HO‐2 and ANXA1/FPR axes to promote polarization of microglia towards M2 type, thereby alleviating inflammation after ischemia. Reduce ROS‐induced oxidative stress by inhibiting the activation of the TRPV1 signaling pathway, upregulating the activity of eNOS, Trx, SOD, and GSH‐Px, or downregulating the expression of NADPH and MDA. During cerebral ischemia, EAP alleviates neuronal apoptosis in the brain by inhibiting the activation of the TRPV1/NF‐κ B signaling pathway, upregulating the GRP78 protein and downregulating the GADD153 protein, and promoting the formation of autophagy pathways by upregulating the expression of TREM2 in neurons. During the reperfusion phase, EAP can inhibit excessive autophagy by downregulating the expression of LC3‐I, LC3‐II, and Beclin‐1, activating the mTORC1 signaling pathway to downregulate p‐ULK1 and FUNDC1, and activating the SIRT1‐FOXO1 signaling pathway to promote p62 protein expression. EAP can protect hippocampal CA1 pyramidal neurons during cerebral ischemia by activating the Wnt/β‐catenin signaling pathway, promoting the expression of 2‐AG, and activating the upregulation of CB1R receptor GluR1, thereby reducing Ca^2^ + influx. EAP can activate CB1R receptors, promote the expression of ERK1/2, C ε PKC, and STAT3 proteins, activate CB2R receptors, adenosine A1 receptors, and AdipoR1 receptors, promote GSK‐3 β phosphorylation, activate delta opioid receptors, μ opioid receptors, and increase the expression of BDNF, reducing brain nerve damage. EAP can improve blood–brain barrier dysfunction by activating Src/PI3K/EphrinB2/EphB4 signaling, upregulating the expression of Claudin‐5 and Occludin, or reducing the expression of ROS, NOX4, MMP‐9, and VEGF.

## 7 DISCUSSION

Whether in cardiovascular disease or cerebrovascular disease, local tissue ischemia caused by vascular occlusion and bleeding is the beginning of a series of cardiovascular and cerebrovascular tissue injuries.^187^, ^188^ Previous studies have shown that tissue damage caused by ischemia can lead to severe damage, loss, and even death of corresponding organs. This process is usually closely related to pathways such as inflammation, oxidative stress, autophagy, and apoptosis.^189^, ^190^, ^191^, ^192^, ^193^, ^194^ At present, a large amount of clinical evidence has proven the therapeutic effect of acupuncture on CCVD. Notably, the intervention of acupuncture not only reflects its therapeutic effect on existing diseases but also can prevent potential diseases, thus more and more researchers believe that using electroacupuncture for early treatment may become a potential prevention strategy.

We systematically reviewed the animal models of basic research in CCVD, the parameters of EAP intervention, and the key molecular mechanisms of its effects (Table 1). In preclinical studies, AMI and MCAO models are commonly used to simulate ischemic injury in CCVD, while MIRI and CI/RI models are used to simulate reperfusion injury caused by blood flow recovery after focal tissue ischemia. These are the main models for studying the pathogenesis of CCVD. The intervention effect of EAP is closely related to the selection of acupoints, the MA of electroacupuncture, depth, intensity, frequency, duration, and waveform. By summarizing the included literature, we found that in the selection of acupoints, EAP and similar acupuncture interventions usually use a single acupoint or a combination of multiple acupoints. In cardiovascular diseases, PC6 is the most commonly used acupoint, while in cerebrovascular diseases, the most commonly used acupoint is GV20. In other parameters, EAP intervention generally lasts for 30 min, the acupuncture depth is mostly between 1.5 and 5 mm, the stimulation intensity is mostly at 1 mA, and the commonly used frequency is low frequency. In terms of molecular mechanisms, the key pathways of EAP in preventing and treating cardiovascular and cerebrovascular diseases are similar. For example, EAP can exert protective effects on cardiovascular and cerebrovascular diseases through anti‐inflammatory reactions, antioxidant stress, antiapoptotic promotion of autophagy, regulation of Ca^2+^ overload, and promotion of vascular regeneration. Of course, the related pathways involved in both have their corresponding specificity. For example, in the prevention and treatment of cardiovascular disease with EAP, the metabolic pathway of glutamate is involved, while in the prevention and treatment of cerebrovascular disease with EAP, the blood–brain barrier homeostasis, and activation of neural receptors promote the release of neurotrophic and protective factors are also involved.

However, the following questions need to be addressed in the future. (1) First, how to choose suitable parameters when using EAP to prevent cardiovascular diseases? This article summarizes the parameters of EAP in the included literature. The intervention time and selection of acupoints are relatively consistent, but it is difficult to unify the parameters such as electroacupuncture frequency, intensity, and needle insertion depth. Therefore, further unification of these parameters is necessary for the clinical promotion of EAP. (2) Second, the pathways involved in EAP are important signaling cascades. Their molecular mechanisms are very complex and closely related to the onset and development of cardiovascular diseases. In most pathways, EAP can affect the course of cardiovascular diseases at multiple levels, but the current literature on how EAP inhibits or activates related pathways is still relatively partial, so more in‐depth and complete research is needed in the future. (3) Lastly, current research on EAP for the prevention of cardiovascular diseases is all animal experiments, and the clinical application of this therapy is still in a blank stage. Therefore, the feasibility of EAP still needs further verification by clinical research.

## AUTHOR CONTRIBUTIONS

JMZ and JJC drafted the manuscript and designed the tables, FYS and ZHC designed the figures and edited the graphic abstract, HTY and XW revised the language and checked the text, TTL reviewed the figures and tables, verified the information, XWL conceived and designed the review, and XZF examined the manuscript. All authors have read and approved the final manuscript.

## FUNDING INFORMATION

This work was supported by the National Key R&D Program of China (2022YFC3500404), the National Natural Science Foundation of China (NSFC, 82105024), and the Tianjin Municipal Health and Wellness Commission (2021056).

## CONFLICT OF INTEREST STATEMENT

The authors declare that this study was conducted in the absence of any commercial or financial relationships that could be construed as potential conflicts of interest.

## Data Availability

Data sharing not applicable to this article as no datasets were generated or analysed during the current study.
